# Supplementary material for: Mapping Bacterial Biofilm on Features of Orthopedic Implants In Vitro
Source: Microorganisms. 2022 Mar 8;10(3):586. doi: 10.3390/microorganisms10030586 (PMC8955338; doi:10.3390/microorganisms10030586)
Supplement: Supplementary file 1 [file microorganisms-10-00586-s001.zip › microorganisms-1517810-supplementary.pdf]

<u>**Supplementary Figures**</u>

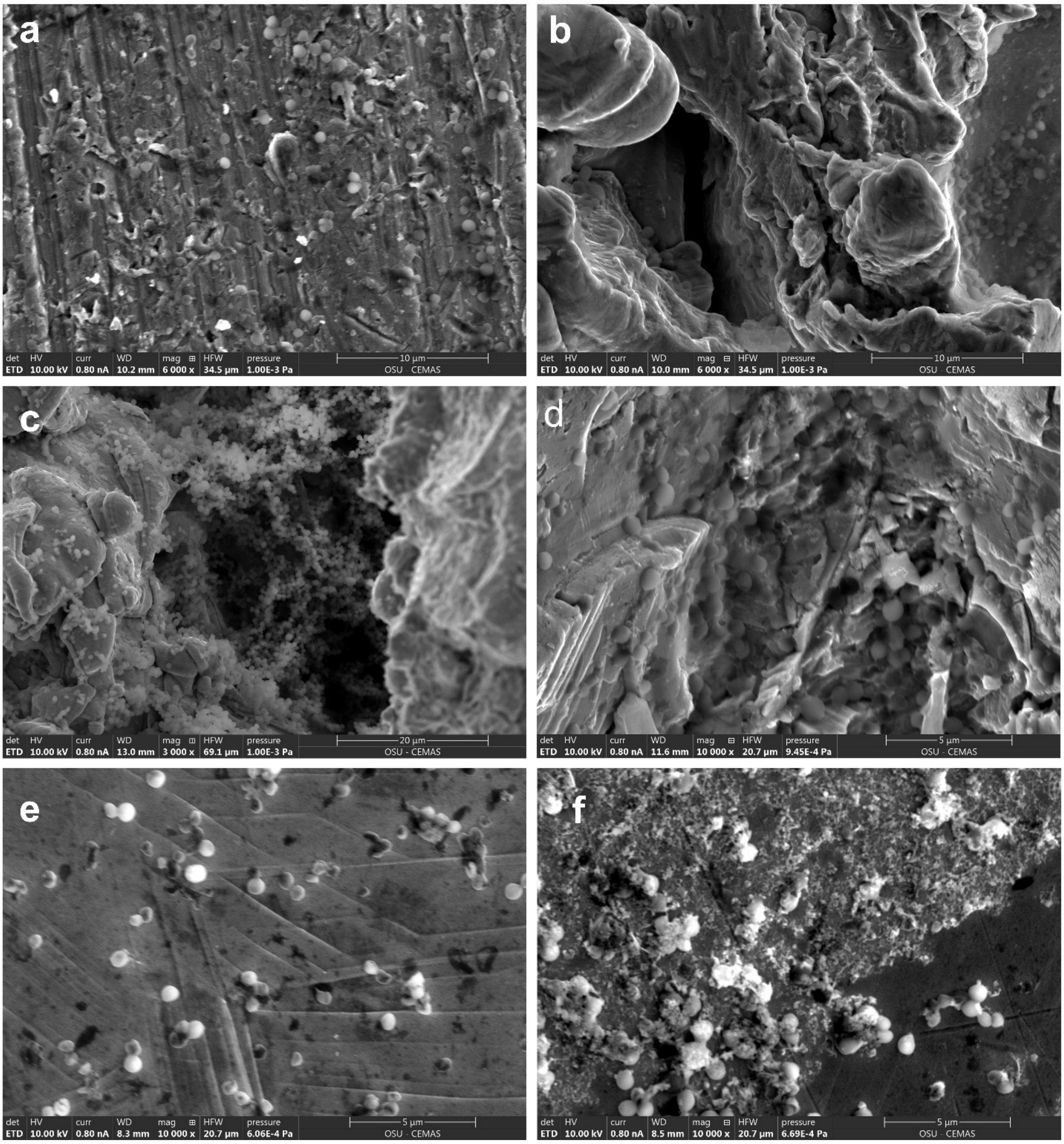

Figure S1. Grayscale images corresponding to Figure 2 in manuscript - SEM images of bacteria adhered to surfaces with different roughness on femoral hip stem and fixation plate. Roughness values (Ra ± σ in μm) on surfaces of a femoral stem were (**a**) 4.14 ± 0.32, (**b**) 71.94 ± 4.85, (**c**) 78.44 ± 2.77, (**d**) 17 ± 0.42, and of fixation plate (**e**) and (**f**) were 4.76 ± 0.51.

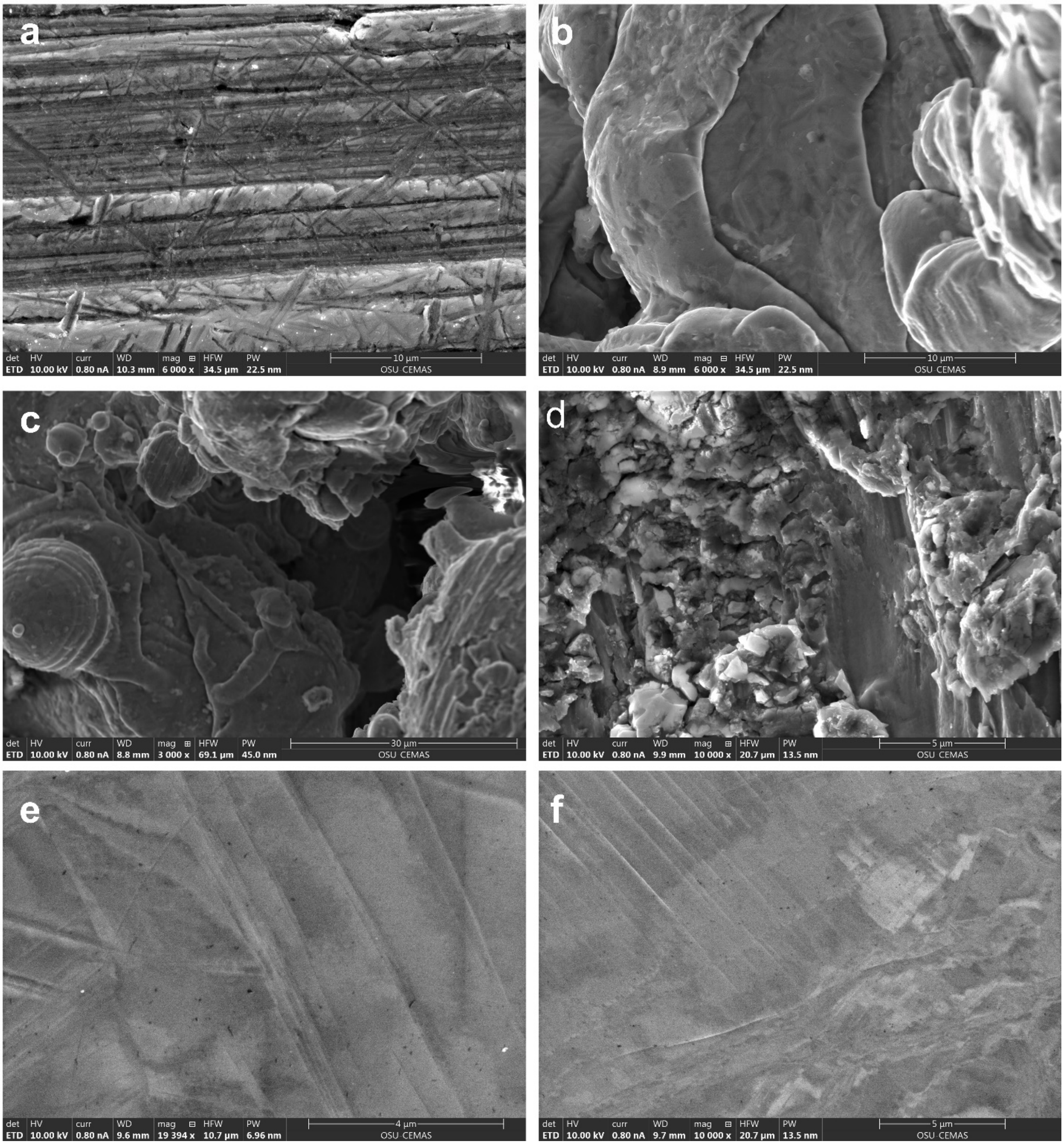

Figure S2. SEM images of implants after cleaning. Roughness values (Ra ± σ in µm) on surfaces of a femoral stem were (**a**) 4.14 ± 0.32, (**b**) 71.94 ± 4.85, (**c**) 78.44 ± 2.77, (**d**) 17 ± 0.42, and of fixation plate (**e**) and (**f**) were 4.76 ± 0.51.

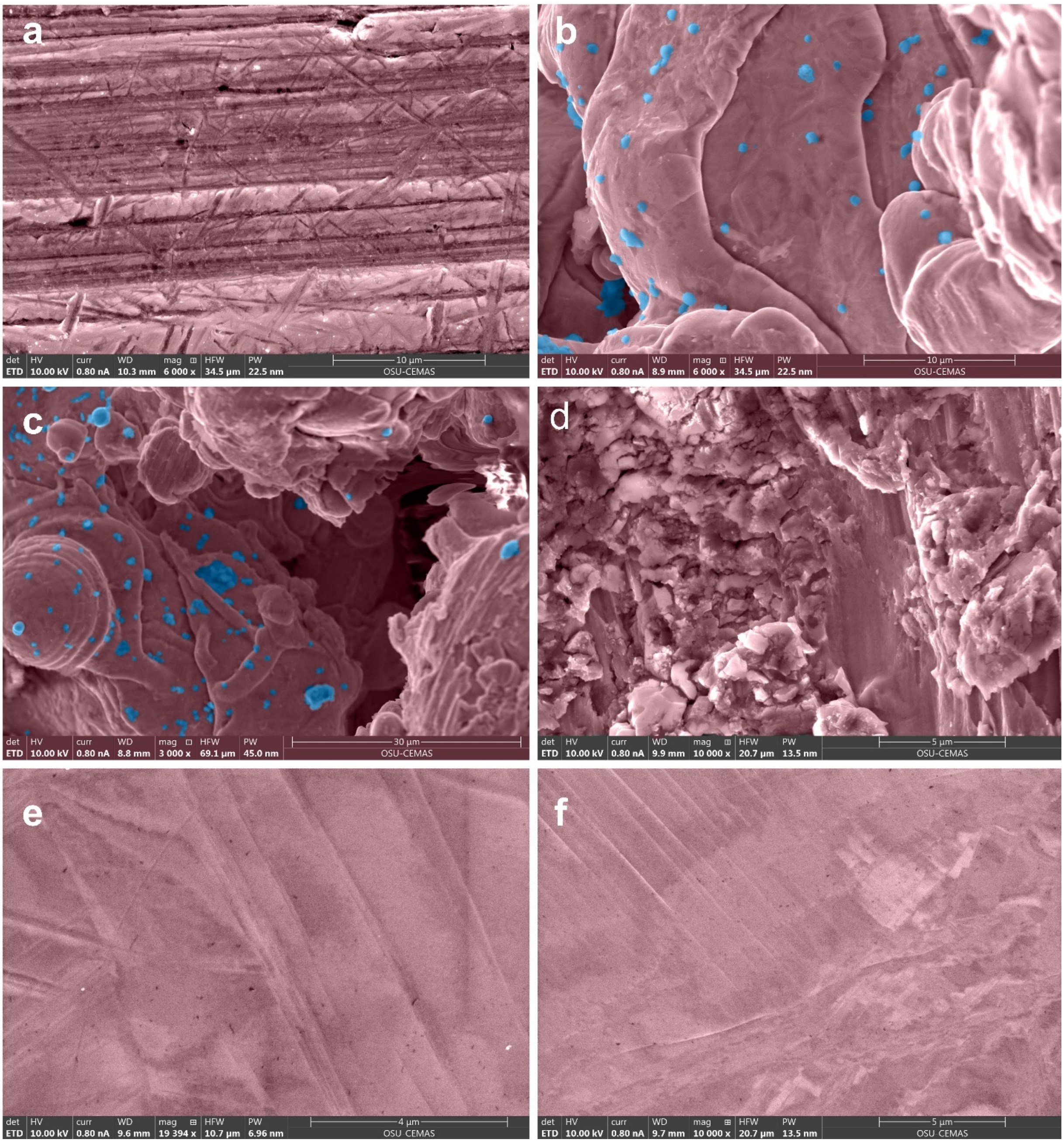

Figure S3. False colored SEM images corresponding to Figure S2.

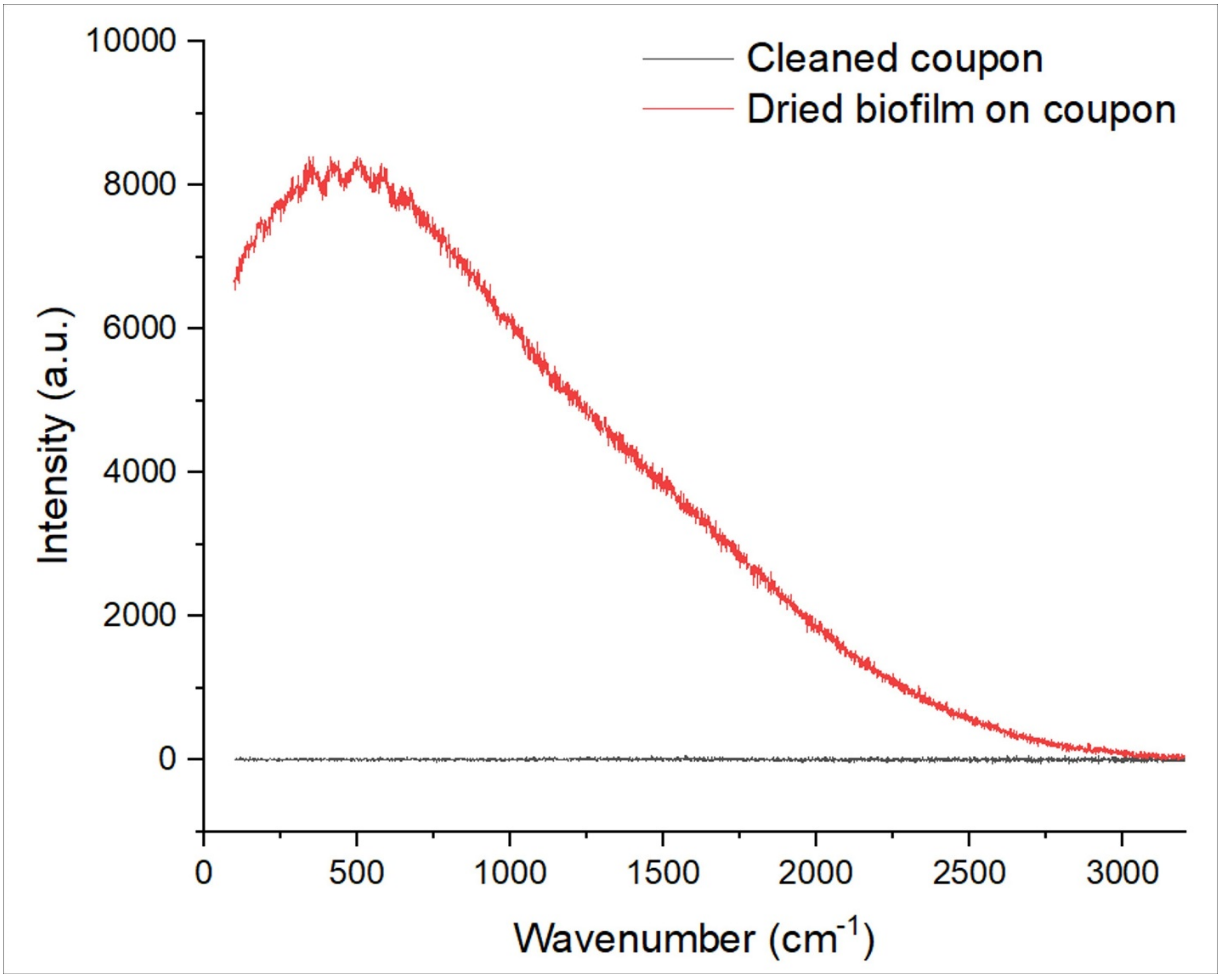

Figure S4. Unprocessed Raman spectra of a stainless-steel coupon after cleaning according to mentioned protocol (black) and biofilm on a cleaned coupon (red). The absence of any signature peaks in the cleaned coupon suggests that there is absence of any organic or soap like materials.
